# Supplementary material for: Biological Activated Sludge from Wastewater Treatment Plant before and during the COVID-19 Pandemic
Source: Int J Environ Res Public Health. 2022 Sep 8;19(18):11323. doi: 10.3390/ijerph191811323 (PMC9517470; doi:10.3390/ijerph191811323)
Supplement: Supplementary file 1 [file ijerph-19-11323-s001.zip › Supplementary Table S1.pdf]

Supplementary Table S1. Comparative analysis regarding the species of microorganisms in activated sludge, identified in this study and those reported in the literature.

Species of microorganisms	References
**Bacteria**: Free bacteria, *Spirilla,* spirochetes, *Zoogloea spp*., Sulphur bacteria, *Sphaerotilus spp., Nocardia, Microthrix,* Type 021N, Type 1701, Type 0041; **Unicelullar sessile**: *Tokophrya spp., Podophrya spp., Opercularia spp., Carchesium spp., Epistylis spp., Vorticella campanula, Vorticella convallaria, Vorticella microst.;* **Unicelullar**: *Coleps spp., Euplotes spp., Aspidisca lynceus, Aspidisca cicada, Chilodonella spp., Litonotus spp., Amphileptus, Tecamoeba, Amoeboids, Paramecium spp., Dexiostoma campyla, Glaucoma spp.,* Zooflagelate, Flagelattes, Wandering cells, Resistance; **Pluricelullar:** Fungi, Rotifers, Nematodes, Insects.	Our study
Peritrichs: *Vorticella convallaria*, *Epistylis entzii*, *Carchesium polypinum*, *Vorticella infusionum*, *Opercularia articulata*; Scuticocilliates: *Dexiotricha tranquilla*, *Cinetochilum margaritaceum*, *Uronema nigricans*, *Cyclidium glaucoma*; Cyrtophorids: *Trochilia minuta*, *Trithigmostoma cucullulus*; Pleurostomatids: *Litonotus lamella*, *Amphileptus pleurosigma*, *Acineria incurvata*, *Litonotus cygnus*; Hypotrichs: *Aspidisca cicada*, *Euplotes affinis*; Peniculins: *Paramecium aurelia complex*, *Paramecium caudatum*; Heterotrichs: *Metopuses*, *Stentor roeselii*.	Martin-Cereceda et al., 2001 [46]
Testaceous rhizopods: *Euglypha sp*., *Arcella sp*., *Trinema sp*., *Cyclopyxis sp*., *Centropyxis sp*.; Nude rhizopods: *Amoeba sp*.; Free swimming ciliates: *Cinetochillum sp*., *Cyclidium sp*., *Uronema sp*.; Large ciliates: *Paramaecium sp*.; Crawling ciliates: *Aspidisca sp*., *Chilodonella sp*., *Oxytricha sp*., Solitary stalked ciliates: *Vorticella sp*., *Acineta sp*.; Colonial stalked ciliates: *Epistylis sp*.; Predator ciliates: *Trachelophyllum sp*., *Amphileptus sp*., *Litonotus sp*.; Rotifers: *Rotaria sp*. and Nemathods.	Ionescu et al., 2015 [51]
Proteobacteria, Bacteroidetes, Chloroflexi, Acidobacteria, *unclassified_f_ Comamonadaceae*, *norank_f_Saprospiraceae*, *Flavobacterium*, *norank_f_ Hydrogenophilaceae*, *Dokdonella*, *Terrimonas*, *norank_f_Anaerolineaceae*, *Tetrasphaera*, *Simplicispira*, *norank_c_Ardenticatenia*, *Nitrospira*, Saprospiraceae, *Flavobacterium*, *Tetrasphaera*.	Xu et al., 2018 [48]
Bacteria: *Acidobacteria*, *Actinobacteria*, *Aminicenantes*, *Armatimonadetes*, *Bacteroidetes*, *Chlamydiae*, *Chlorobi*, *Chloroflexi*, *Cyanobacteria*, *Deferribacteres*, *Elusimicrobia*, *Fibrobacteres*, *Firmicutes*, *Fusobacteria*, *Gemmatimonadetes*, *Gracilibacteria*, *Hydrogenedentes*, *Ignavibacteriae*, *Latescibacteria*, *Microgenomates*, *Nitrospirae*, *Parcubacteria*, *Peregrinibacteria*, *Planctomycetes*, *Proteobacteria*, RBG-1[*Zixibacteria*], SR1[Absconditabacteria], *Saccharibacteria*, *Spirochaetae*, *Synergistetes*, TM6[*Dependentiae*], *Verrucomicrobia*.	Yang et al., 2020 [49]
Protozoa and Rotifers: *Diatoms (Bacillariophycophyta)*, *Paramecium spp*., *Vorticella spp*., *Nematodes*, *Trypanosomes*, *Motile algal filaments*; Bacteria: *Pseudomonas spp*., *Escherichia coli*, *Staphylococcus aureus*, *Klebsiella spp*., *Alcaligenes spp*., *Sphaerotilus natans*, *Beggiatoa spp*.; Fungi: *Aspergillus spp*., Geotrichum spp.	Joshi et al., 2013 [53]
Anaerolineaceae uncultured, Hydrogenophilaceae uncultured, *Dechloromonas*, Saprospiraceae uncultured, *Planctomyces*, *Alphaproteobacteria* unclassified, *Betaproteobacteria* unclassified, Rhodocyclaceae unclassified, *Nitrospira*, *Rhizobium*, *Thiothrix*, *Pseudomonas*, *Zoogloea*, Hyphomicrobiaceae uncultured, *Rhizobia*, Hyphomicrobiaceae, *Alphaproteobacteria*, *Zoogloea*.	Chen, et al., 2017 54]
Bacteria: *Chryseobacterium gregarium*, *Staphylococcus epidermidis*, *Stenotrophomonas acidaminiphila*, *Brevibacillus parabrevis*, *Cloacibactéruim normanense*, *Pseudomonas veronii*, *Acinetobacter soli*, *Acinetobacter parvus*.	Nouha et al., 2015 [55]
Protozoa; Metazoa: Gastrotrichs, Nematodes, Monogononta rotifers; Naked amoeba; Testacea; Predatory ciliates; Attached ciliates; Crawling ciliates; Flagellates; *Cochlipodium* sp., *Arcella* sp., *Peranema* sp., *Epistylis chrysemidis*, *Microthorax pusillus*, *Metacystis* sp., *Thuricola* sp., *Opercularia* spp., *A. cicada*, *Chilodonella* sp., *Vorticella* sp., *V. convallaria*, *H. discolor, M. pusillus, Peranema* sp., *Monogononta*, *Bdelloidea*, *Thuricola* sp., *Metacystis* sp., *Plagiocampa rouxi.*	Sobczyk et al., 2021 [47]
