# Supplementary figures and images for: Biological Activated Sludge from Wastewater Treatment Plant before and during the COVID-19 Pandemic
Source: Int J Environ Res Public Health. 2022 Sep 8;19(18):11323. doi: 10.3390/ijerph191811323 (PMC9517470; doi:10.3390/ijerph191811323)

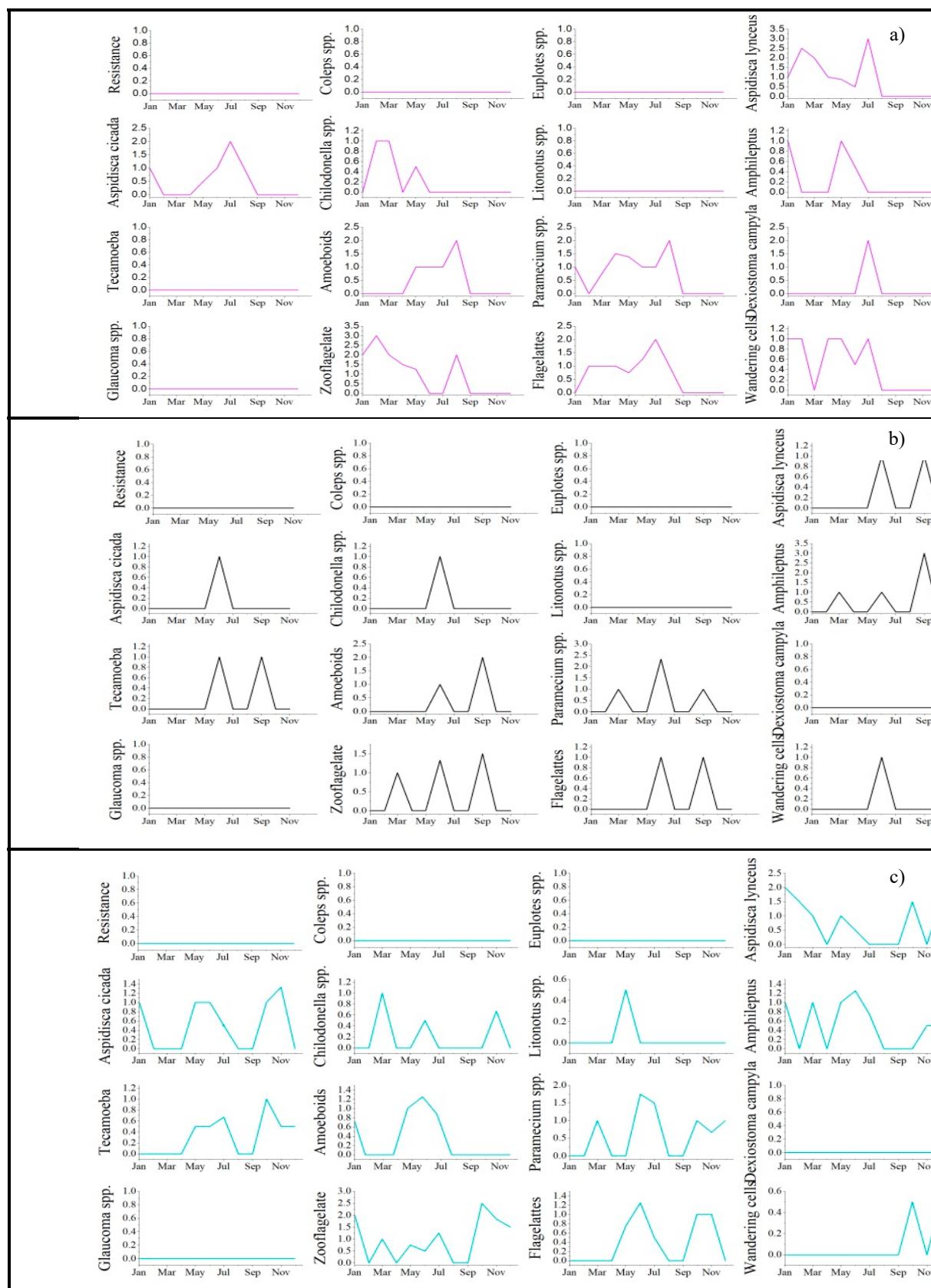

**Supplementary Figure S1.** Unicellular free abundance in the three years of study: (a) 2019; (b) 2020; (c) 2021.

Supplement: Supplementary file 1 [file ijerph-19-11323-s001.zip › Supplementary Figure S1.pdf]
